# Stent-assisted Embolization of a Giant Aneurysm of the Middle Cerebral Artery Using Small and Large Coils (Penumbra Coil 400)

**DOI:** 10.7759/cureus.762

**Published:** 2016-08-31

**Authors:** Edgar Gerardo Ordónez-Rubiano, Christian Von-Diemling, William Cortes-Lozano, Nelson Oswaldo Lobelo-Garcia

**Affiliations:** 1 Fundación Universitaria de Ciencias de la Salud, Hospital de San Jose/Hospital Infantil Universitario de San José; 2 Department Allg. Innere Medizin, Klinik Hirslanden; 3 Neurosurgery, Fundación Universitaria de Ciencias de la Salud, Hospital de San Jose/Hospital Infantil Universitario de San José; 4 Neuroradiology, Fundación Universitaria de Ciencias de la Salud, Hospital de San Jose/Hospital Infantil Universitario de San José

**Keywords:** endovascular, giant aneurysm, stent, embolization, large coils, coiling

## Abstract

Giant aneurysms have been treated with endovascular approaches like general, balloon-assisted and stent-assisted coiling, and flow diverter stent-assisted techniques. Few cases have been reported to be treated with both normal and large coils. Despite the mass effect, an adequate revascularization has been reported. An initial use of these coils is being reported in the current study. This is a case which has been successfully treated using a stent-assisted coiling with both small and large coils i.e., Penumbra Coil 400 (Penumbra, Inc., Alameda, California).

## Introduction

The middle cerebral artery (MCA) is the most common location for giant aneurysms. About 15% of all MCA aneurysms are giant aneurysms [1]. A giant aneurysm is defined as an aneurysm with a diameter of more than 25mm. Large giant aneurysms of the MCA are rare lesions. Their natural history, as well as the etiopathogenesis, do not remain completely elucidated. The incidence of a giant aneurysm is reported to be 6.1% [2]. There is still a controversial discussion if clipping or coiling MCA aneurysms are superior or better for the patient, and often there is no ideal solution. Some authors prefer clipping the aneurysm because of the possibility of intraoperative hematoma evacuation if needed [3]. Lately, published studies have shown better outcomes when treating an aneurysm with a Penumbra 400 Coil in comparison to other conventional coils because of its higher packing density and lower number of coils per aneurysm, while achieving similar occlusion rates. Another advantage of these coils seems to be the cost-effectiveness [4, 5]. We describe the case of a giant aneurysm of the MCA, which was treated successfully at our institute with Penumbra Coil 400. 

## Case presentation

**Figure 1:**
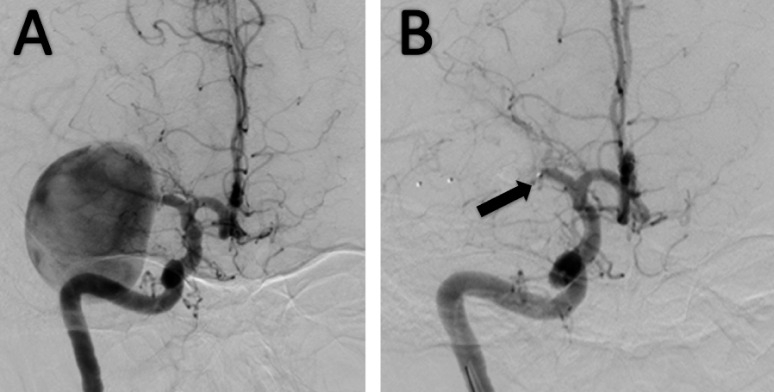
Pre-embolization Catheter Angiography Images (A) There is a 5x5x5cms giant Middle Cerebral Artery aneurysm, with flush-filling to the superolateral wall. Perforating arteries are denoted proximal to the aneurysm. (B) The ballon for the Ballon-assisted Occlusion Test is depicted (arrow).

A 27-year-old woman presented to the emergency department with nausea, vomiting, and global headache. The admission of non-contrast CT of the head showed a rounded hyperdense lesion in the left Sylvian fissure, without evidence of acute hemorrhage or associated hydrocephalus. A subsequent magnetic resonance angiogram (MRA) was performed showing a giant aneurysm of the left MCA. A catheter angiography was further performed which demonstrated a giant aneurysm of the M1 segment of the left MCA, with a distant flow to M3 and M4 segments, and collaterals coming from the left anterior cerebral artery (ACA). Before deciding whether to occlude the vessels or not, a balloon-assisted occlusion test (BOT) with a Scepter balloon in the proximal ostium of the aneurysm was performed, which resulted in a transitory facial paralysis. The parent vessel occlusion could not be performed. Therefore, it was decided to perform stent-assisted coil embolization of the aneurysm using both small and large coils. (Figure 1) A right femoral artery entry was used with an 8F-introducer, through which a 0.88 Neuronmax catheter was entered up to the right internal carotid artery (ICA). Subsequently, a distal support Vasco microcatheter was catheterized with a 0.014 microwire to the segment of the artery distal to the aneurysm. A Slim-px microcatheter was used to access the proximal M1 segment of the right MCA. A 3D-DSA reconstruction was performed and used as a railroad map. 

**Figure 2:**
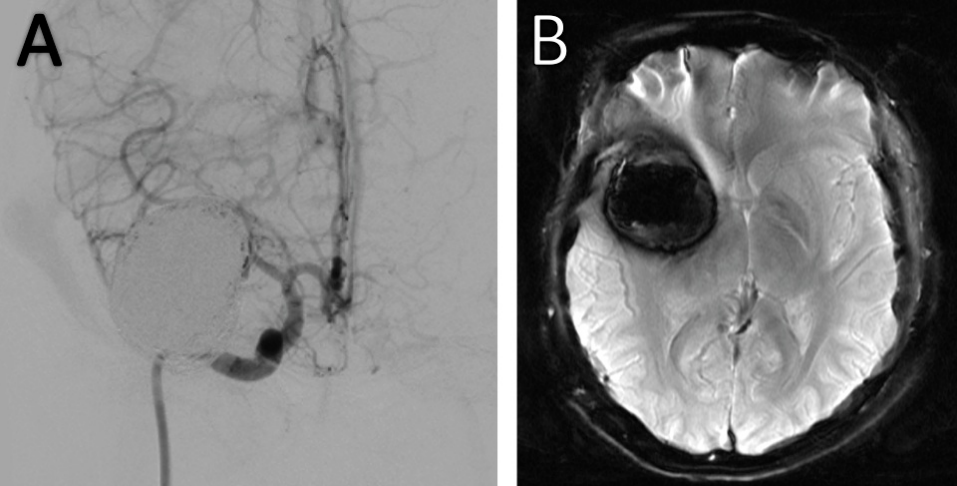
Post-embolization MRI and Catheter Angiography Images (A) Catheter angiography and (B) Susceptibility-Weighted Magnetic Resonance Imaging post-coiling one-year-control demonstrating adequate embolization with thrombosis and without partial filling of the aneurysm.

Posteriorly, an Enterprise stent was successfully introduced, and a complete recanalization was achieved. After the placement of the stent, a total of 36 coils (26 conventional and seven large coils) were used. Subsequent one and two-year follow-up MRAs and angiograms showed adequate packaging (Figure 2). The patient presented occasional headaches, but there was no hemorrhage or other complications related to the treatment of the aneurysm.

## Discussion

When the BOT is tolerated, the parent vessel occlusion is the primary therapy for a large and giant aneurysm with the exception of the basilar tip. Surgical bypass should be reserved only for cases where an aneurysm is located intradurally and has a high potential of re-rupture and cannot be treated by parent arterial occlusion or endovascular approaches [2]. Here we describe the treatment of a giant right middle cerebral artery aneurysm using small and large coils (Penumbra 400 coil). An estimation of how many conventional coils would be needed is challenging and depends on the size and length of coils used. We chose this coil because recent studies in comparison with conventional coils have shown that the  Penumbra Coil 400 provides a higher packing density with a lower number of coils per aneurysm while achieving similar occlusion rates. Furthermore, it seems to be more cost-effective [4, 5]. Achieving adequate packing density during aneurysm treatment is often challenging. We know from studies which investigated the packing attenuation (inserted coil volume/aneurysm volume), that the majority of aneurysms that are packed more than 25% stay stable [6], therefore it is critical to achieve a high packing density. 

## Conclusions

This case demonstrates an adequate treatment of a giant MCA aneurysm with a stent-assisted coiling with Penumbra Coil 400. However, the mass effect of embolization material could be a cause of the residual headaches. 
